# Reducing the Complexity of Complex Gene Coexpression Networks by Coupling Multiweighted Labeling with Topological Analysis

**DOI:** 10.1155/2013/676328

**Published:** 2013-10-07

**Authors:** Alfredo Benso, Paolo Cornale, Stefano Di Carlo, Gianfranco Politano, Alessandro Savino

**Affiliations:** ^1^Department of Controls and Computer Engineering, Politecnico di Torino, 10129 Torino, Italy; ^2^Consorzio Interuniversitario Nazionale per l'Informatica, 11029 Verres, Italy; ^3^Department of Agriculture, Forest and Food Sciences, Università degli Studi di Torino, 10124 Torino, Italy

## Abstract

Undirected gene coexpression networks obtained from experimental expression data coupled with efficient computational procedures are increasingly used to identify potentially relevant biological information (e.g., biomarkers) for a particular disease. However, coexpression networks built from experimental expression data are in general large highly connected networks with an elevated number of false-positive interactions (nodes and edges). In order to infer relevant information, the network must be properly filtered and its complexity reduced. Given the complexity and the multivariate nature of the information contained in the network, this requires the development and application of efficient feature selection algorithms to be able to exploit the topological characteristics of the network to identify relevant nodes and edges. This paper proposes an efficient multivariate filtering designed to analyze the topological properties of a coexpression network in order to identify potential relevant genes for a given disease. The algorithm has been tested on three datasets for three well known and studied diseases: acute myeloid leukemia, breast cancer, and diffuse large B-cell lymphoma. Results have been validated resorting to bibliographic data automatically mined using the ProteinQuest literature mining tool.

## 1. Introduction

Systems biology typically uses networks to model and discover emergent properties among genes, proteins, and other relevant biomolecules referred to specific phenotypes or diseases.

Theoretical studies have revealed that biological networks share many features with other types of networks such as computer or social networks. They enable the application of several mathematical and computational methods of the graph theory to biological studies [1, 2]. The computational analysis of biological networks has therefore become increasingly used to mine the complexity of cellular processes and signaling pathways.

Many types of biological networks do exist, depending on the information associated to their nodes and edges. In general, they can be classified as directed or undirected networks [3]. In directed networks, nodes are molecules, while edges indicate causal biological interactions among nodes (e.g., transcription and translation regulations [4]). Instead, in undirected networks, an edge indicates a shared property, such as sequence similarity [5], gene coexpression [6–9], protein-protein interaction [10], or term cooccurrence in the scientific literature [11–13].

Undirected gene coexpression networks, coupled with efficient computational algorithms and complemented by the literature mining, may represent a valuable instrument to identify relevant information (e.g., biomarkers) for a particular disease. However, coexpression networks built from experimental expression data are in general large highly connected networks with an elevated number of false-positive interactions (nodes and edges). In order to infer relevant information, the network must be properly filtered and its complexity reduced. Given the complexity and the multivariate nature of the information contained in the network, this requires the development and application of efficient feature selection algorithms to be able to exploit the network topological characteristics.

Several feature selection algorithms have been proposed in the literature: some in the context of generic machine learning techniques [14] and others more specifically designed to work with transcriptome data [15–21]. Following Saeys et al. [21], feature selection methods fall in three main categories, namely, (1) filters, (2) wrappers, and (3) embedded methods.

Filters assess the relevance of features by looking at the intrinsic properties of the data. They do not consider any learning or classification model [19]. Filtering techniques easily scale to very high-dimensional datasets. They are computationally simple and fast, and they are independent of any machine learning model to be applied after filtering. However, a common disadvantage of filtering methods is that most of the proposed techniques are univariate [19], which is a major limitation for transcriptome data analysis. In fact, genes tend to work according to complex gene regulatory networks, and their expression profiles are therefore highly correlated. To overcome this limitation, multivariate filtering techniques to some extent incorporating feature dependencies have been introduced [22, 23]. Unfortunately, they are in general slower and less scalable than univariate techniques, thus preventing their application on genome-wide transcriptome data.

Wrappers [24] and embedded approaches [17] differ from filters since they are designed to work with specific machine learning and classification models. The main difference between the two groups of approaches is that in embedded approaches feature selection is built into the classifier, while wrappers work together with the classifier but are not part of it. In general, these approaches are able to improve the classification accuracy. However, different classification models may highlight different sets of relevant genes, and sometimes genes that might be biologically representative are discarded by the classification model.

In this paper, we exploit gene coexpression networks and network topological analysis to implement an efficient multivariate filtering algorithm attempting to reduce the size of the network under analysis and to identify sets of genes, which have a biological relevance for a given disease. In particular, a multiweighted coexpression network, is built on top of collected expression data in order to efficiently represent correlations among genes in high-dimensional expression datasets. A topological analysis algorithm is then applied to the coexpression network to identify regions of the network with interesting topological properties that may highlight relevant genes for the modeled phenomenon.

We tested the proposed approach on a set of microarray experiments for three well-studied diseases and compared the obtained list of relevant genes with a bibliometric correlation list of genes retrieved resorting to the ProteinQuest [25] tool. Statistical analysis on the obtained results highlighted that the proposed approach is able to strongly reduce the size of the analyzed coexpression networks, while keeping genes that are highly correlated with the target diseases in the scientific literature.

## 2. Methods and Materials

The network filtering approach proposed in this paper includes two computational steps designed to

1. organize the expression data into a multiweighted coexpression network that is able to better highlight relationships among nodes compared to single-weighted networks, and
2. analyze the multi-weighted network in order to identify regions of the network with interesting topological properties that may highlight relevant genes for the modeled phenomenon.

### 2.1. Multiweighted Coexpression Networks

Let us consider a dataset of expression data (e.g., microarray data) for a large number of DNA sequences tested under two different conditions (e.g., healthy tissue versus diseased tissue).

We organize the dataset in the form of a gene expression matrix GEM : *N* × *M* → *e*_*i*,*j*_ ∈ *ℜ* with rows representing samples and columns representing genes. Each element of the matrix provides the differential expression level *e*_*i*,*j*_ of gene *g*_*j*_ (column *j*) in sample *s*_*i*_ (row *i*) under the two tested conditions. Among the different ways to compute differential expression, in this paper, we exploit the (binary) logarithm of the ratio between the absolute expression of the gene in the two tested conditions (log ratio). Log ratios tend to be normally distributed [26] and enable to easily normalize expression levels from different samples using standard score (*z* score) normalization [27]:

(1) $$\begin{array}{c} e_{i,j}=\frac{\text{lo}{\text{g}}_{2}\left(eC1_{i,j}/eC2_{i,j}\right)-\mu_{i}}{\sigma_{i}}, \end{array}$$

where *eC*1_*i*,*j*_ and *eC*2_*i*,*j*_ represent the absolute expression of gene *g*_*j*_ in sample *s*_*i*_ under the conditions *C*1 and *C*2, and *μ*_*i*_ and *σ*_*i*_ denote the mean and the standard deviation of log ratios of all genes within sample *s*_*i*_.

Normalized expression data can be used to build a single coexpression network enabling to easily identify genes that are coexpressed within an experiment. For our analysis we exploit a multi-weighted coexpression network (MWNET) that assigns multiple weights to the network edges to identify different forms of coexpression among genes.

An MWNET is an undirected weighted graph MWNET = (*V*, *E*, *W*), where

(i) vertexes *g*_*j*_ ∈ *V* represent genes that are differentially expressed in at least one of the available samples;
(ii) edges *E*⊆*V* × *V* connect pairs of vertexes (*g*_*j*_, *g*_*k*_) and represent genes that are coexpressed in at least a sample;
(iii) a weight function *W* assigns to each edge a vector of weights:
  (2) $$\begin{array}{c} W:\left(g_{i},g_{j}\right)\in E\mapsto {\vec{w}}_{i,j}=\left(w_{i,j}^{\text{OO}},w_{i,j}^{\text{OS}},w_{i,j}^{\text{SO}},w_{i,j}^{\text{SS}}\right). \end{array}$$

A gene *g*_*j*_ is considered differentially expressed in sample *s*_*i*_ if |*e*_*i*,*j*_| > *ε*; that is, its differential expression is greater than a given threshold required to filter residual noise in the data. If the differential expression of a gene is positive, it means the gene is overexpressed in condition *C*1 compared to condition *C*2. We denote the gene as overexpressed. In the opposite condition, that is, negative differential expression, the gene is instead denoted as silenced.

Exploiting the concept of overexpressed and silenced genes, each edge of the network is labeled with four weights associated to the four combinations of expression conditions; the pair of genes connected by the edge may assume the following: (1)  *w*_*i*,*j*_^OO^ both genes over-expressed, (2)  *w*_*i*,*j*_^OS^ gene *g*_*i*_ overexpressed and *g*_*j*_ silenced, (3)  *w*_*i*,*j*_^SO^ gene *g*_*i*_ silenced and *g*_*j*_ over-expressed, and (4)  *w*_*i*,*j*_^SS^ both genes silenced. Each weight counts how many times the pair of genes assumes the selected state in the set of samples composing the dataset.

**Figure 1:**
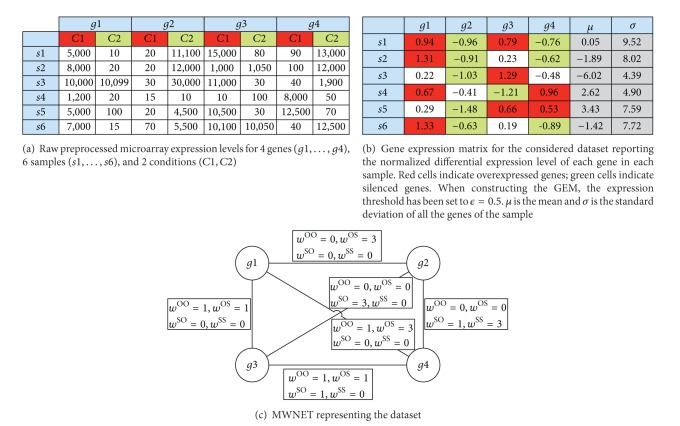
Example of construction of a MWNET. Expression data in this example are not from real experiments. They are simply used to show the process required to construct a MWNET starting from raw expression data.

Figure 1 shortly summarizes the steps required to construct an MWNET starting from raw preprocessed expression data.

Multi-weighted coexpression networks are the first original contribution of this paper and are able to provide interesting insights about gene relations. Network edges highlight relationships among genes with the weights providing a measure of the strength and the type of the relation. Moreover, by looking at the different edges, additional information can be inferred. Genes connected with edges with high *w* ^OO^/*w* ^SS^ score (i.e., both genes over-expressed or silenced) may underly a biological behavior in which the two genes enhance/silence each other, which is a common motif in biological networks [28, 29]. Similarly genes connected with edges with high *w* ^OS^/*w* ^SO^ may identify genes connected with negative loops, which again is a common motif observed in several biological networks [29–31].

### 2.2. Network Filtering

Multi-weighed coexpression networks built from experimental expression data are in general complex highly connected networks that contain an elevated number of false-positive interactions (edges).

The weights assigned to each edge represent a valuable information to remove interaction with low informative content.

Let us consider two candidate genes A and B connected by an edge in a network built from a set of *N* samples. A uniform weight distribution on the edge (e.g., ${\vec{w}}_{A,B}=[N/4,N/4,N/4,N/4]$) identifies a low informative interaction since genes show differentiated behaviors among the different samples. Differently, if the weights are polarized toward one of the four behaviors (e.g., ${\vec{w}}_{A,B}=[N,0,0,0]$), the informative content of the edge increases.

This consideration is exploited to build a filtering mechanism for the selected network. A relevance score (*R*_*g*_*i*__) defined according to the following equation is assigned to each gene *g*_*i*_ of the network according to the following equation:

(3) Rgi=1N∑∀j ∣ ∃(gi,gj)∈E[1−min⁡(w→i,j)max⁡(w→i,j)]·[2·σ(w→i,j)N] ∀i∈[1,M].

Equation (3) tries to assign high scores to genes that are connected to their neighbors with strong polarization. If two genes are connected with almost uniform weight distribution, the minimum and the maximum weights are similar and the first term of the equation tends to zero thus lowering the score. In all other cases, the more the edges are weighted with a not uniform distribution, the more their score increases.

The relevance score introduced by (3) is used to filter the list of nodes and edges of the network thus reducing its complexity. Setting a threshold on the acceptable relevant scores allows us to remove low relevant genes and their related edges, thus obtaining a filtered list of genes and a reduced network ready for further analyses aiming at identifying interesting network motifs [4, 31–34].

The proposed relevance score is defined in order to highlight those genes that manifest expression changes between the two considered conditions *C*1 and *C*2 in a significant number of samples of the considered data set. The quality and the numerosity of the available data are therefore the key issues to properly compute this score. This is even more critical when samples collected for a given disease include different phenotypes. If a given phenotype is not properly represented in the dataset, the risk of ranking its specific markers with low relevant scores becomes high, with the risk of losing important biological information during the filtering process that could provide unexpected leads for biologic or therapeutic insights. Nevertheless, this is a common drawback of all machine learning and statistical methods that can only be mitigated by increasing the number of collected samples and carefully selecting them in order to be representative for the considered phenomenon.

Moreover, the threshold used to filter the network is a good instrument to deal with the risk of loosing significant genes. A tight threshold will in general filter the presence of different phenotypes. It enables to obtain a smaller network representative of the common properties of the considered disease, regardless of the specific phenotypes. Instead, by relaxing the threshold, also genes that are informative in a reduced set of samples will be included in the filtered network. Researchers may look at this low ranked genes as candidates nodes that are able to highlight specific phenotypes, and therefore conduct further experimental investigations.

## 3. Results and Discussion

In our experimental design, we tested the proposed network filtering algorithm on three coexpression networks obtained from expression data for three well studied and documented diseases with available on-line datasets.

1. *Acute Myeloid Leukemia Dataset (AML)*: peripheral-blood samples or bone marrow samples of intermediate-risk AML with a normal karyotype [35]. This dataset includes 14 samples with 43,196 spots (45 K technology) obtained from microarray data available at the Gene Expression Omnibus (GEO), accession number GSE426. The complete list of selected samples is available in Table 1.
2. *Breast Cancer* (BC): samples of predominantly advanced primary breast tumor [36]. This dataset includes 20 samples with 9,216 spots (9 K technology) obtained from microarray data available at the Gene Expression Omnibus (GEO), accession number GSE3281. The complete list of selected samples is available in Table 2.
3. *Diffuse Large B-Cell Lymphoma Dataset (DLCL)*: a set of samples from patients with diffuse large B-cell lymphoma, the most common subtype of non-Hodgkin's lymphoma downloaded from a larger dataset of experiments aiming at performing Lymphoma classification [37, 38]. This dataset includes 51 samples with 9,216 spots (9 K technology) obtained from microarray data available at the Gene Expression Omnibus (GEO), accession number GSE60. The complete list of selected samples is available in Table 3.

Samples have been downloaded from the cDNA Stanford Microarray database [39]. All genes without a valid *Unigene ID* have been discarded. The normalized differential expression for each gene has been computed according to (1) considering the CH1I_MEAN and the CH2I_MEAN mean intensity channels available for each microarray as absolute expression level of each gene, and *ε* = 0 (1-folding). Since old microarray technologies often used spots duplication, during the network generation we considered as expressed those genes differentially expressed in at least one of their replica on the microarray.

The filtering process has been executed on the three considered datasets applying a quite relaxed threshold of 0.5 on the relevance score of (3).

Table 4 shows the aggregated results in terms of number of genes before and after filtering, highlighting the relevant reduction ratio. The full list of identified genes is instead provided as Supplementary Material to this paper available online at http://dx.doi.org/10.1155/2013/676328 (global_citation_summary.xlsx file—BC Filtered Genes, AML Filtered Genes and DLCL Filtered Genes sheets).

Validation of the proposed filtering algorithm has been performed by comparing the list of filtered genes with data mined from the available scientific literature. It is worth to mention here that this validation phase does not aim at identifying new markers for the considered diseases. By selecting three diseases that have been intensively studied and documented, we aim at confirming how the proposed method is able to identify relevant genes from raw expression profiles that are widely confirmed in the available literature. In order to rely on a large literature dataset, rather then performing manual searches, the literature has been mined resorting to the ProteinQuest bibliography data mining tool [25].

ProteinQuest is an advanced text-mining tool that exploits the web services offered by PubMed to perform advanced semantic searches of scientific papers. It searches for biological terms (e.g., diseases, proteins, genes, miRNAs, etc.) in titles and abstracts as well as in all image captions of all papers stored in Medline. Image captions are extracted, from free full-text articles, using the BFO Java library (http://bfo.com/) on the PDF version of the scientific papers [40]. ProteinQuest is capable of inheriting the PubMed MeSH terms indexing. ProteinQuest text-mining tool searches, in the abstracts and figure captions of all identified publications, for terms belonging to a manually curated protein dictionary based on Entrez MeSH terms. Common ambiguities in the terminology are resolved using a multiple search for more than one alias, as well as the cooccurrence of specific words, which can deny or force the tagging process.

ProteinQuest has been first used to manually refine the obtained lists of filtered genes in order to remove generic oncogenes that cannot be specifically ascribed to a selected disease. For instance, the AML's filtered genes contain TP53, a tumor suppressor protein crucial in multicellular organisms for cell cycle regulation [41]. TP53 is a clear example of a generic oncogene not specifically marking a particular cancer disease. To enhance the specificity of the network to the selected disease, it has been manually removed after filtering.

As a preliminary validation, we searched using ProteinQuest for all genes that have been cocited with one of the three available diseases submitting the query: “Leukemia, Myeloid, Acute” or “Breast Neoplasm” or “Lymphoma, Large B-Cell, Diffuse.” The query is designed not to lose genes included in subsets such as phenotypes, and subtypes, by resorting to the most general keywords available for each disease. The query selected 10,488 genes extracted from 269,641 analyzed publications, and returned for each gene and for each disease the number of detected co-citations (global_citation_summary.xlsx file—Citation Data sheet—Columns A–D). For each disease, we sorted the list of 10,488 selected genes by their decreasing citation count, assigning to each gene a disease gene citation ranking (global_citation_summary.xlsx file—Citation Data sheet—Columns H–J). Top ranked genes are highly cocited with the disease. Finally, for each disease, we marked those genes present in the list of filtered genes obtained by our filtering algorithm. (global_citation_summary.xlsx file—Citation Data sheet—Columns E–G).

**Figure 2:**
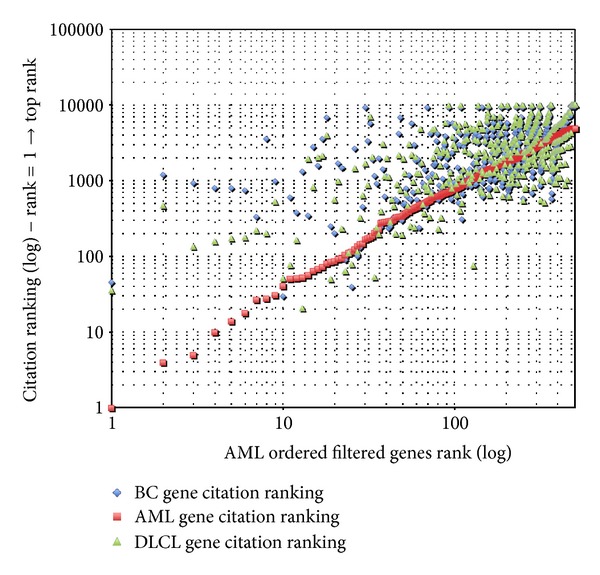
AML preliminary bibliometric validation.

Starting from these citation data, Figure 2 summarizes the preliminary validation performed for the AML dataset. Citation data have been filtered to select the AML filtered genes, only. Resulting data have been sorted by the AML citation rank and the citation rank of each selected gene for each of the three diseases has been plotted. The three citation ranks for each gene are always vertically aligned.

Looking at the left side of  Figure 2, one can notice that top ranked genes for the AML datasets (i.e., high number of co-citations between the gene and the disease) have, in general, a lower rank for the other two disease (i.e., lower number of co-citations), thus giving an indication that the genes selected by our algorithm have a higher bibliometric correlation with the selected disease. This selectivity decreases moving to lower ranked genes. It is worth to remember here that filtering was performed with a quite relaxed threshold that on the one hand limits the selectivity of the filtering process but, on the other hand, preserves low ranked genes that might be important to characterize specific phenotypes of the considered disease. This is, for example, the case for the diffuse large B-cell lymphoma. The considered dataset includes samples for two well-known phenotypes of this disease: (1) GC and (2) activated [37]. Thanks to the relaxed threshold, genes CD38, BCL7A, BCL6, MYB, PI3, CD2, CASP10 that are well known to be differentially expressed across the two phenotypes, even if not at the top of the ranked list, have been preserved in the filtered network, thus enabling to preserve this relevant information across the filtering process.

**Figure 3:**
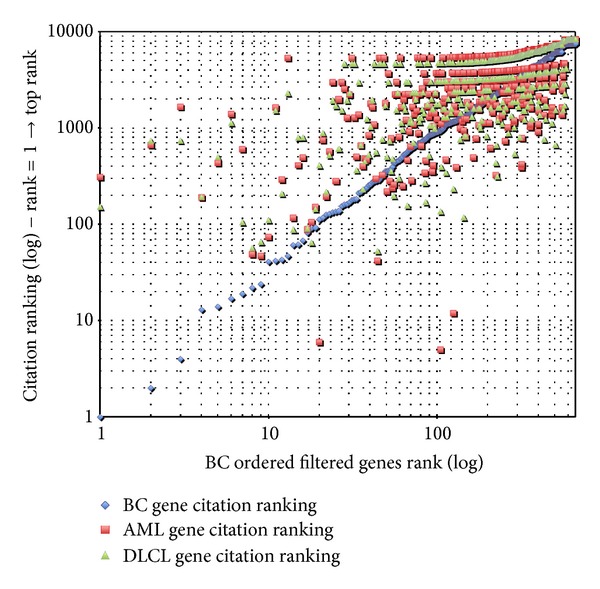
BC preliminary bibliometric validation.

**Figure 4:**
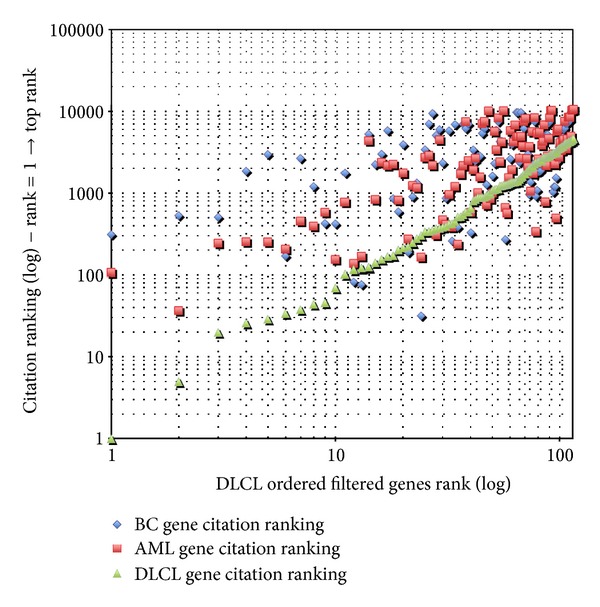
DLCL preliminary bibliometric validation.

 A similar trend is also confirmed looking at the citation data for the remaining two diseases reported in Figures 3 and 4.

In order to perform a more solid statistical validation through the use of bibliometric data, we executed a set of queries on ProteinQuest to understand if, given a disease, the set of genes selected by our algorithm is highly cocited with the disease while showing low citation count with the other diseases. As an example, Algorithm 1 shows the query executed to search for citation relevance of AML genes with AML related publications. The query searches for papers in which at least one of the selected genes is cocited with the AML disease and not cocited either with BC or DLCL diseases. The query produces, for each gene, the number of papers in which the selected condition is respected.

 Data obtained from the execution of these ProteinQuest queries have been aggregated in Table 5 that reports, for each group of filtered genes, the cumulative citation count for each disease. The full set of data returned by the execution of each query and used to construct Table 5 is available in the disease_citation_heatmaps.xlsx file provided as additional material of this paper. By construction, each query guarantees that citations obtained on each column of the table are disjoint.

We analyzed data reported in Table 5 using SAS software (version 9.1.3; SAS Institute, Cary, NC, USA). In order to find relationship between genes groups and diseases, frequencies of citations have been analyzed. For each group of genes, pairwise differences among diseases have been performed using the FREQ procedure. Furthermore, Bonferroni adjustment of the obtained *P* values has been carried out with the MULTTEST procedure.

The chi-square test reveals that citations frequencies among diseases (AML, DLCL, and BC) significantly differ by groups of selected genes (*χ*_(4)_^2^ = 64,897.4; *P* < 0.0001). Considering the first group of selected genes, the frequency of citations referred to AML (49.79%) significantly differs from both DLCL (*χ* ^2^ = 13,662; *P* < 0.0001) and BC (*χ* ^2^ = 743.5; *P* ≤ 0.0001) citations (12.28% and 38%, resp.). Similarly, the proportion among citations in the second group of selected genes highlights significant differences between DLCL citations frequency (54.47%) and AML citations (13%; *χ* ^2^ = 6,496.5; *P* < 0.0001) and between DLCL and BC citations (32.53%; *χ* ^2^ = 1,112.1; *P* < 0.0001). Finally, greater frequency of citations has been observed in the third group of genes when comparing BC (83.92%) over AML (11.72%; *χ* ^2^ = 3,206.2; *P* < 0.0001) and over DLCL (4.36%; *χ* ^2^ = 233,024; *P* < 0.0001). The obtained results on frequencies of citations support the ability of the algorithm in selecting appropriate genes group according to the selected disease.

## 4. Conclusions

In this paper we proposed a multi-weighted network topology, and a related algorithm for its analysis, that is able to filter complex coexpression networks obtained from gene expression data in order to reduce the network size and to retain only those genes and those interactions that are potentially related to a selected disease.

The algorithm has been tested on three public datasets for well-known studied diseases proving its high efficiency in reducing the complexity of the network. Moreover, to show that the filtering process is able to keep nodes, which are relevant for a particular disease, a validation campaign that resorts to bibliometric data mined through the ProteinQuest tool is presented.

The proposed approach represents a valuable starting point to reduce the complexity of complex biological networks in order to perform further analyses aiming at identifying interesting network motifs.

## Supplementary Material

Global citation summary.xlsx: This file integrates citation data for the three identified list of genes and correlates them with the related disease. These aggregated data enable to rank genes in a disease based on their citation count and to analyze the capability of our tool in selected genes that are highly cited in the literature with the related disease.
Disease_citation_heapmap.xlsx: This file reports raw citation data obtained using ProteinQuest to understand if, given a disease, the set of genes selected by our algorithm is highly cocited with the disease while showing low citation count with the other diseases. Data reported in this file are therefore obtained by running the query available in Algorithm 5 of the paper for the identified list of genes for the three considered diseases.

## Figures and Tables

**Algorithm 1:**
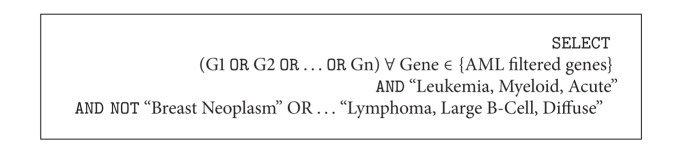
ProteinQuest query example to obtain citation data for the AML dataset.

**Table 1:** List of samples for the AML dataset. Samples are cDNA 45 K array technology.

Sample number	GEO accession number	Experiment name
1	GSM6259	AML 13
2	GSM6266	AML 28
3	GSM6281	AML 21
4	GSM6284	AML 112
5	GSM6309	AML 32
6	GSM6317	AML 20
7	GSM6318	AML 111
8	GSM6319	AML 18
9	GSM6275	AML 1
10	GSM6285	AML 25
11	GSM6292	AML 105
12	GSM6311	AML 24
13	GSM6335	AML 16
14	GSM6337	AML 114

**Table 2:** List of samples for the BC data-set. Samples are cDNA 9 K array technology.

Sample number	GEO accession number	Experiment name
1	GSM73756	BC-16 versus NF (svi114)
2	GSM73784	808A versus NF (svi060)
3	GSM73706	107B versus NF (svi032)
4	GSM73726	110B versus NF (svi033)
5	GSM73727	111A versus NF (svi034)
6	GSM73732	111B versus NF (svi035)
7	GSM73734	114A versus NF (svi037)
8	GSM73736	115B versus NF (svi038)
9	GSM73783	710A versus NF (svi056)
10	GSM73764	118B versus NF (svi041)
11	GSM73786	123B versus NF (svi043)
12	GSM73704	206A versus NF (svi045)
13	GSM73708	214B versus NF (svi048)
14	GSM73709	305A versus NF (svi049)
15	GSM73738	308B versus NF (svi050)
16	GSM73776	402B versus NF (svi052)
17	GSM73777	406A versus NF (svi053)
18	GSM73779	708B versus NF (svi054)
19	GSM73697	805A versus NF (svi058)
20	GSM73699	807A versus NF (svi059)

**Table 3:** List of samples for the DLCL data-set. Samples are cDNA 9 K array technology.

Sample number	GEO accession number	Experiment name
1	GSM2035	DLCL-0047
2	GSM2036	DLCL-0042
3	GSM1958	DLCL-0040
4	GSM1959	DLCL-0036; OCT
5	GSM2037	DLCL-0035
6	GSM1994	DLCL-0034
7	GSM2038	DLCL-0033
8	GSM1995	DLCL-0032
9	GSM1996	DLCL-0031
10	GSM1997	DLCL-0030
11	GSM1998	DLCL-0029
12	GSM1960	DLCL-0028
13	GSM1999	DLCL-0027
14	GSM2039	DLCL-0026
15	GSM2040	DLCL-0025
16	GSM2000	DLCL-0024
17	GSM2001	DLCL-0023
18	GSM2041	DLCL-0021
19	GSM2043	DLCL-0019
20	GSM2044	DLCL-0018
21	GSM2045	DLCL-0016
22	GSM2047	DLCL-0014
23	GSM2048	DLCL-0013
24	GSM2049	DLCL-0012
25	GSM2050	DLCL-0011
26	GSM2051	DLCL-0010
27	GSM2052	DLCL-0009
28	GSM2053	DLCL-0008
29	GSM2055	DLCL-0006
30	GSM2056	DLCL-0005
31	GSM2058	DLCL-0003
32	GSM2059	DLCL-0002
33	GSM2060	DLCL-0001
34	GSM1965	DLCL-0052 *||*lc4b060
35	GSM1967	DLCL-0041 *||*lc4b061
36	GSM1968	DLCL-0039 *||*lc4b039
37	GSM1969	DLCL-0037 *||*lc4b036
38	GSM2072	DLCL-0034 *||*lc8n109
39	GSM1972	DLCL-0033 *||*lc4b034
40	GSM2073	DLCL-0032 *||*lc8n110
41	GSM2074	DLCL-0031 *||*lc8n108
42	GSM2016	DLCL-0028 *||*lc7b025
43	GSM2077	DLCL-0027 *||*lc8n095
44	GSM1974	DLCL-0025 *||*lc4b059
45	GSM2078	DLCL-0024 *||*lc8n096
46	GSM2079	DLCL-0023 *||*lc8n098
47	GSM1976	DLCL-0015 *||*lc4b063
48	GSM1977	DLCL-0011 *||*lc4b030
49	GSM1978	DLCL-0010 *||*lc4b053
50	GSM1979	DLCL-0009 *||*lc4b027
51	GSM1982	DLCL-0002 *||*lc4b033

**Table 4:** Aggregated filtering results for the three considered data-sets.

	Original number of genes	Filtered number of genes	Reduction ratio
AML	39,028	505	98.70%
BC	7,531	662	91.20%
DLCL	6,826	115	98.31%

**Table 5:** Citations of groups of filtered genes according to the disease.

Group of genes	Disease			Total
	AML	DLCL	BC	
Number 1 AML filtered genes	23,248	5,741	17,769	46,758
	(49.72), [63.68]	(12.28), [28.56]	(38.00), [17.56]	

Number 2 DLCL filtered genes	2,470	10,347	6,180	18,997
	(13.00), [6.77]	(54.47), [51.47]	(32.53), [6.11]	

Number 3 BC filtered genes	10,787	4,015	77,223	92,025
	(11.72), [29.55]	(4.36), [19.97]	(83.92), [76.33]	

Total	36,505	20,103	101,172	

( ): percentage in rows; [ ]: percentage in columns.
